# Mendelian randomization study of lithocholate sulfate mediating the effect of MMP-1 on ischemic stroke

**DOI:** 10.1097/MD.0000000000048010

**Published:** 2026-03-13

**Authors:** Yuyu Wei, Jialong Tian, Xiaojun Pang, Huai Chen, Xuhong Jin, Zibin Zhang

**Affiliations:** a Department of Neurosurgery, Zhejiang Hospital of Integrated Traditional Chinese and Western Medicine, Hangzhou, Zhejiang, China.

**Keywords:** circulating inflammatory cytokines, disease-associated metabolites, ischemic stroke, mediation analysis, Mendelian randomization

## Abstract

This study aimed to investigate the potential causal relationship between the inflammatory factor matrix metalloproteinase-1 (MMP-1) and ischemic stroke (IS) using Mendelian randomization (MR), and to examine whether the gut microbiota-derived metabolite lithocholate sulfate (LSL) mediates this association. We performed a 2-sample MR analysis to assess the causal effect of genetically predicted MMP-1 levels (based on 14,744 individuals) on IS risk (39,818 cases and 2,71,817 controls). Genetic instruments were single nucleotide polymorphisms associated with the exposures, sourced from published genome-wide association studies. The causal direction was verified through reverse MR analysis. Furthermore, a multivariable MR analysis was conducted to quantify the mediating effect of LSL on the pathway from MMP-1 to IS. Genetically predicted higher MMP-1 levels were significantly associated with an increased risk of IS (inverse variance weighted odds ratio: 1.085, 95% confidence interval [CI]: 1.031–1.142, *P* = .002). In contrast, reverse MR analysis provided no evidence for a causal effect of IS on MMP-1 levels (inverse variance weighted odds ratio: 1.034, 95% CI: 0.954–1.120, *P* = .420). The mediation analysis revealed a significant indirect effect mediated by LSL, with an estimated proportion of 0.0071 (95% CI: 0.0027–0.0168). This MR study provides genetic evidence that elevated MMP-1 is causally associated with an increased risk of IS. Furthermore, we identify LSL as a protective mediator in this pathway. Our findings propose a novel MMP-1 → LSL → IS axis, suggesting a compensatory interaction where a pro-inflammatory signal may upregulate a protective metabolite. This highlights LSL as a potential target for future research into stroke mechanisms and prevention.

## 1. Introduction

Ischemic stroke (IS) is characterized by the blockage of cerebral arteries due to various reasons, leading to a situation where the cerebral blood flow is insufficient to meet the needs of brain tissue and ultimately resulting in brain tissue death due to ischemia and hypoxia. Common causes of IS include atherosclerotic plaque detachment, atrial fibrillation, thrombosis,^[1]^ and smoking.^[2]^ Representing the most prevalent type of cerebrovascular disease, IS accounts for ~70% to 80% of these cases.^[3]^ It primarily affects patients with focal neurological deficits, such as hemiplegia, sensory impairment, aphasia, ataxia, and insomnia.^[4]^ While mild cases often have a favorable prognosis, severe cases can be life-threatening.^[5]^ Prompt and effective treatment, monitoring, and rehabilitation programs are multifaceted necessities for managing IS.^[6]^ In addition to the common clinical causes, circulating inflammatory cytokines^[7]^ and 1400 metabolites implicated in IS have gradually emerged as research focal points.

Jing Hua Zhao et al conducted a genome-wide protein quantitative trait locus study on 91 plasma proteins from 14,824 subjects. They demonstrated that the inflammatory response is closely related to the onset and progression of numerous diseases. In other words, circulating inflammatory cytokines are also related to IS.^[8]^

Metabolites, a category of small molecules involved in physiological metabolism, are prone to disturbances from both in vitro and in vivo factors, including genetics, sleep, exercise, diet, gut microbiota, and the environment. By studying metabolites, we can gain insights into disease risk and identify potential treatment targets. Yiheng Chen et al used Mendelian randomization (MR) analysis to examine the causal effects of 1091 blood metabolites and 309 metabolite ratios on various diseases.^[9]^ Through genome-wide association studies (GWAS) of metabolites, they revealed their roles in common diseases and provided pertinent evidence for subsequent treatment.

To the best of our knowledge, no studies have reported a certain inflammation factor through metabolites implicated in human disease that mediate the occurrence of IS. MR analyses, a recent research hotspot, can eliminate a variety of confounding factors. The primary feature of MR is the use of genetic variants as instrumental variables (IVs) to investigate the role of exposure factors in disease outcomes from a genetic perspective. We separately explored the causal association between 1400 inflammatory factors and IS,^[3]^ further investigating the role of the metabolite as an intermediary factor through MR. This is the first study to propose and demonstrate that metabolites are intermediary factors involved in the regulation of IS by inflammatory factors.^[10]^

## 2. Materials and methods

### 2.1. Data collection

In this study, we investigated the causal relationship between circulating inflammatory factors and IS via 2-sample bidirectional MR. We conducted a multi-sample MR analysis among inflammatory factors, metabolites, and IS to identify statistically significant metabolites. As in most MR analyses, single nucleotide polymorphisms (SNPs) served as the primary IVs for subsequent investigations.^[11]^

#### 2.1.1. Ischemic stroke

We sourced genetic data on IS from the GWAS database of the FennGenn consortium,^[12]^ which includes 39,818 cases and 2,71,817 controls. The specific download site is https://storage.googleapis.com/finngen-public-data-r9/summary_stats/finngen_R9_C_STROKE.gz.

#### 2.1.2. Inflammatory factors

We obtained genetic data on 91 circulating inflammatory cytokines from the GWAS database (GCST90274758-GCST90274848).^[8]^ In the MR analysis, with circulating inflammatory cytokines as exposure factors and IS as outcome events, matrix metalloproteinase-1 (MMP-1; GCST90274826) was ultimately identified as a significant inflammatory factor, including 14,744 samples. Specific data URL: http://ftp.ebi.ac.uk/pub/databases/gwas/summary_statistics/

#### 2.1.3. Metabolites implicated in human diseases

Summary data for 1400 metabolites implicated in human diseases were sourced from a published article: Genomic Atlas of the plasma metabolome prioritizes metabolites implicated in human diseases (*Published in final edited form as: Nat Genet. 2023 January 01; 55 (1): 44–53. doi:10.1038/s41588-022-01270-1*).^[9]^ Genetic data for these 1400 metabolites were retrieved from the GWAS database (GCST90199621-GCST90201020). All GWAS data were derived from distinct sample groups, ensuring no data overlap.

Specific data URL: http://ftp.ebi.ac.uk/pub/databases/gwas/summary_statistics/.

### 2.2. Data analysis

1. Core MR assumptions and IV selection

This 2-sample MR study is based on 3 core assumptions: Relevance assumption: the genetic IVs must be strongly associated with the exposure (e.g., MMP-1 or lithocholate sulfate [LSL]).^[2]^ Independence assumption: The IVs must not be associated with any confounders of the exposure-outcome relationship.^[3]^ Exclusion restriction assumption: the IVs must affect the outcome (IS) only through the exposure, with no alternative biological pathways.

Guided by these assumptions, the following inclusion and exclusion criteria were applied for IV selection:

Inclusion criteria (for selecting SNPs strongly associated with the exposure):

1. SNPs with genome-wide significance (*P* < 5 × 10^−8^) were prioritized.
2. If an insufficient number of SNPs met the above threshold, the significance level was relaxed to *P* < 5 × 10^−5^ to increase the number of available genetic instruments, a practice widely adopted in prior MR studies to ensure statistical power.

Exclusion criteria (to ensure IV independence and exclusion restriction):

1. SNPs in high linkage disequilibrium were pruned to ensure independence (parameters: window size = 10,000 kb, *r*^2^ < 0.001).
2. SNPs with an *F*-statistic < 10 were excluded to mitigate weak instrument bias.
3. A PhenoScanner database (v2) search was performed to identify and exclude SNPs significantly associated with known major IS risk factors (e.g., hypertension, atrial fibrillation, diabetes, dyslipidemia) or potential confounders affecting LSL metabolism (e.g., liver disease, gut microbiota-related disorders).
4. Palindromic SNPs were excluded, and allele alignment was performed to ensure consistency of effect alleles across exposure and outcome datasets.

1. Relationship between MMP-1 and IS

We performed 3 sequential 2-sample MR analyses to systematically test our hypotheses. Assessing the causal effect of MMP-1 on IS. This step aimed to establish genetic evidence for MMP-1 as a risk factor for IS.

**Figure 1.**
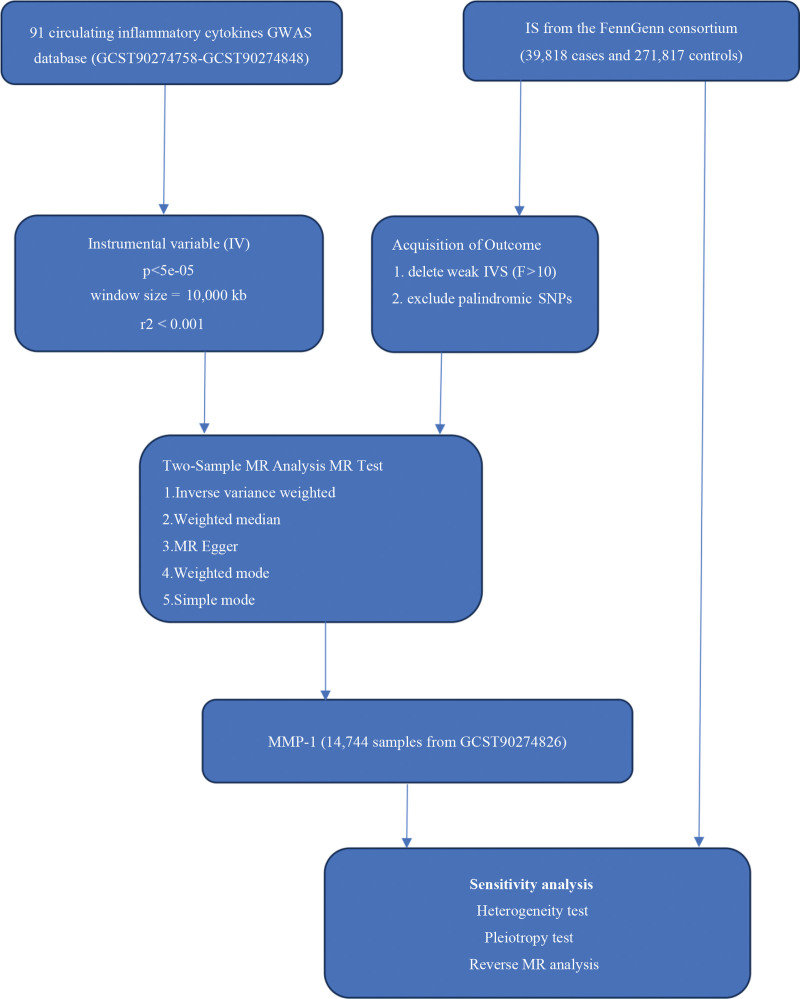
Schematic representation of the study. We conducted MR analysis on 2 samples for each circulating inflammatory cytokine versus IS to evaluate the causal relationship between 91 circulating inflammatory cytokines and IS. Following the identification of the significant inflammatory factor MMP-1, we completed the reverse MR analysis of MMP-1 and IS. GWAS = genome-wide association studies, IS = ischemic stroke, IV = instrumental variable, MMP-1 = matrix metalloproteinase-1, MR = Mendelian randomization.

**Figure 2.**
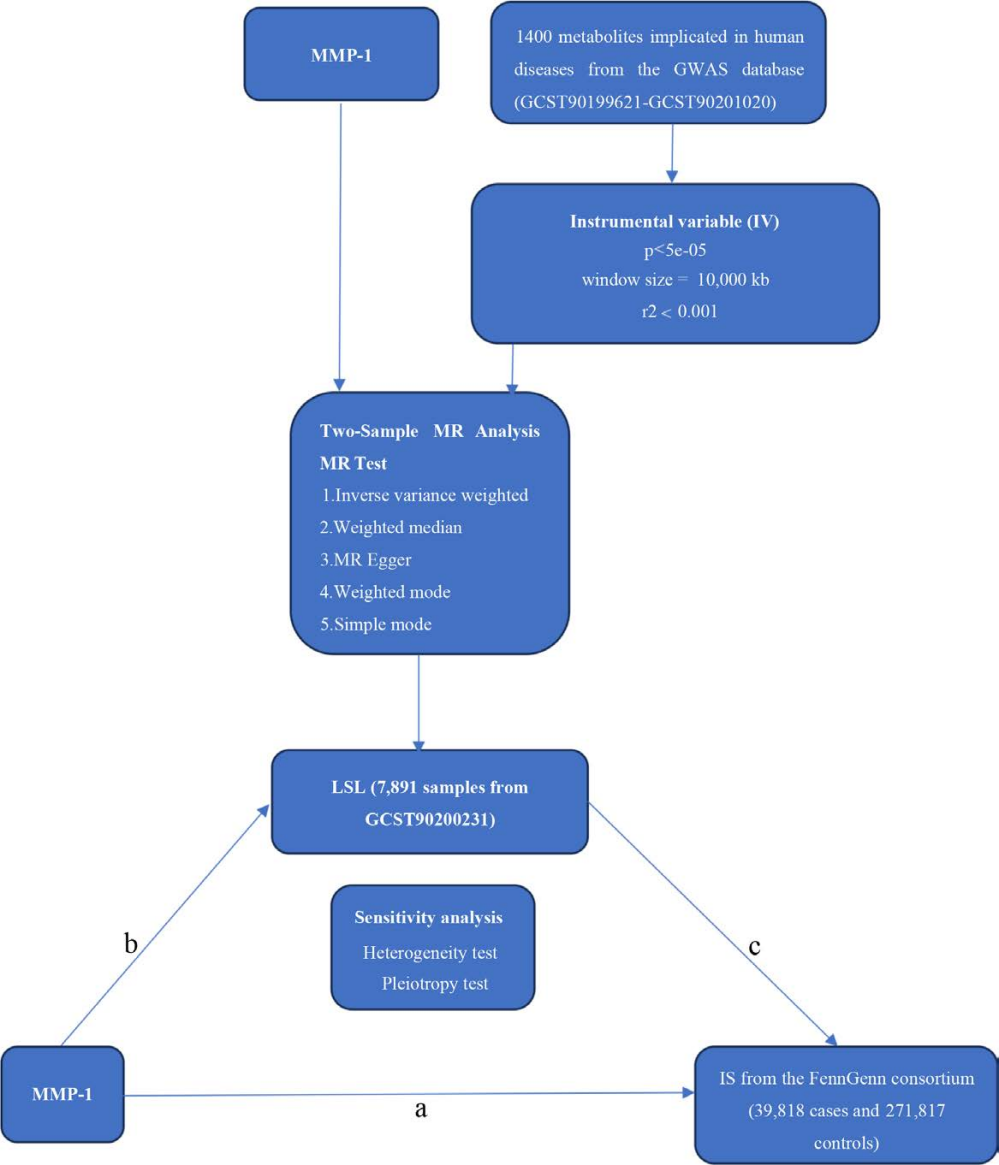
Schematic representation of the study. We performed a 2-sample MR analysis to assess the causal relationship between MMP-1 and IS. We calculated the LSL-mediated effect of MMP-1 on IS using multistep MR. The total effect (*a*) was 0.0817, *b* = 0.1081, *c* = −0.0655; the indirect effect was 0.00708 (*b* × *c*); and the direct effect (*d*) was 0.0888 (*a* − *b* × *c*). IS = ischemic stroke, LSL = lithocholate sulfate, MMP-1 = matrix metalloproteinase-1, MR = Mendelian randomization.

Initially, we conducted a 2-sample MR analysis for each circulating inflammatory cytokines versus IS to evaluate the causal relationship between 91 circulating inflammatory cytokines and IS. Following the identification of the significant inflammatory factor MMP-1, we completed the reverse MR analysis of MMP-1 and IS (Fig. 1). Finally, we confirmed a unidirectional causal relationship between MMP-1 and IS^[13]^ (Table 1, Fig. 1). We designated the results of the previous analysis as total effect a (Fig. 2). The overall effect was divided into direct (without intermediary effect, defined as *d*) and indirect (intermediary effect) effects.^[10]^ The direct effect was calculated as the total effect minus the indirect effect.

**Table 1:** Results of the correlation analysis among ischemic stroke, MMP-1, and IS.

		SNP	OR 95% CI	*P*
MMP-1 on IS	MR-Egger	25	1.1166 (1.0142–1.2293)	.0345
	Weighted median	25	1.0970 (1.0271–1.1716)	.0059
	Inverse variance weighted	25	1.0852 (1.0311–1.1421)	.0017
IS on MMP-1	MR-Egger	41	1.0465 (0.9274–1.1808)	.4653
	Weighted median	41	1.0798 (0.9676–1.2050)	.1702
	Inverse variance weighted	41	1.0335 (0.9540–1.1197)	.4198
MMP-1 on LSL	MR-Egger	27	1.2196 (1.0362–1.4355)	.0248
	Weighted median	27	1.0650 (0.9361–1.2118)	.3387
	Inverse variance weighted	27	1.1142 (1.0219–1.2148)	.0142
LSL on IS	MR-Egger	22	0.9886 (0.9009–1.0848)	.8114
	Weighted median	22	0.9198 (0.8631–0.9802)	.0100
	Inverse variance weighted	22	0.9366 (0.8989–0.9759)	.0018

Heterogeneity and pleiotropy in MR analyses.

CI = confidence interval, IS = ischemic stroke, LSL = lithocholate sulfate, MMP-1 = matrix metalloproteinase-1, MR = Mendelian randomization, OR = odds ratio, SNP = single nucleotide polymorphism.

1. Relationship between MMP-1 and LSL levels

Assessing the causal effect of MMP-1 on LSL levels. This step investigated whether the inflammatory marker (MMP-1) exerts an upstream regulatory effect on the candidate protective metabolite (LSL).

We then performed an MR analysis of MMP-1 and 1400 metabolites implicated in human diseases, defining the results analyzed here as effect *b*.

1. Relationship between LSL and IS

Assessing the causal effect of LSL on IS. This step independently tested for a genetic causal association between LSL itself and IS risk.

Subsequently, the statistically significant metabolites (LSL) and IS were subjected to further MR analysis, with the results analyzed here defined as effect *c*.

1. LSL-mediated mediation between MMP-1 and IS

Provided that steps A–D demonstrated significant causal associations in the hypothesized directions (MMP-1↑ → IS risk↑; MMP-1↑ → LSL↑; LSL↑ → IS risk↓), we proceeded to quantify the mediating proportion of LSL in the MMP-1–IS causal pathway using a mediation analysis framework based on multivariable MR.

The indirect effect was the product of outcome *b* and outcome *c* (Fig. 2). We computed the LSL-mediated mediating effect (indirect effects) of MMP-1 on IS via multistep MR.^[10]^ In summary, our final results were total effect (*a*), indirect effect (*b* × *c*), and direct effect (*d* = *a* − *b* × *c*; Table 1, Fig. 2).

1. Primary statistical methods for MR analysis

MR analyses were conducted using R software (version 4.3.1, http://www.r-project.org) with the “two-sample MR” package (version 0.6.2). Which provides the core functions for implementing MR analyses and data harmonization. To ensure robust causal inference, we employed the MR-pleiotropy residual sum and outlier (MR-PRESSO) package and assessed assumptions using standard tests for pleiotropy and heterogeneity. We reviewed pertinent literature and employed a PhenoScanner search to assess all known phenotypes associated with the genetic tools considered in our analysis.^[10]^ There were few overlapping data or confounding IVS between exposure and outcome studies. Wald ratios for the causal effects of each SNP were combined by meta-analysis using inverse variance weighted (IVW), which is currently a relatively precise method for evaluating the results of MR analysis. The IVW method was selected as our primary analytical approach because it provides the most statistically efficient estimate of the causal effect under the assumption that all genetic instruments are valid (i.e., no horizontal pleiotropy). Two complementary analytical strategies, MR-Egger and weighted median, were utilized to support the IVW analysis results. These methods were chosen as key sensitivity analyses. The MR-Egger method can detect and provide an estimate corrected for overall directional pleiotropy, while the weighted median method yields a robust causal estimate even if up to 50% of the genetic instruments are invalid, thus testing the resilience of our primary findings.

**Figure 3.**
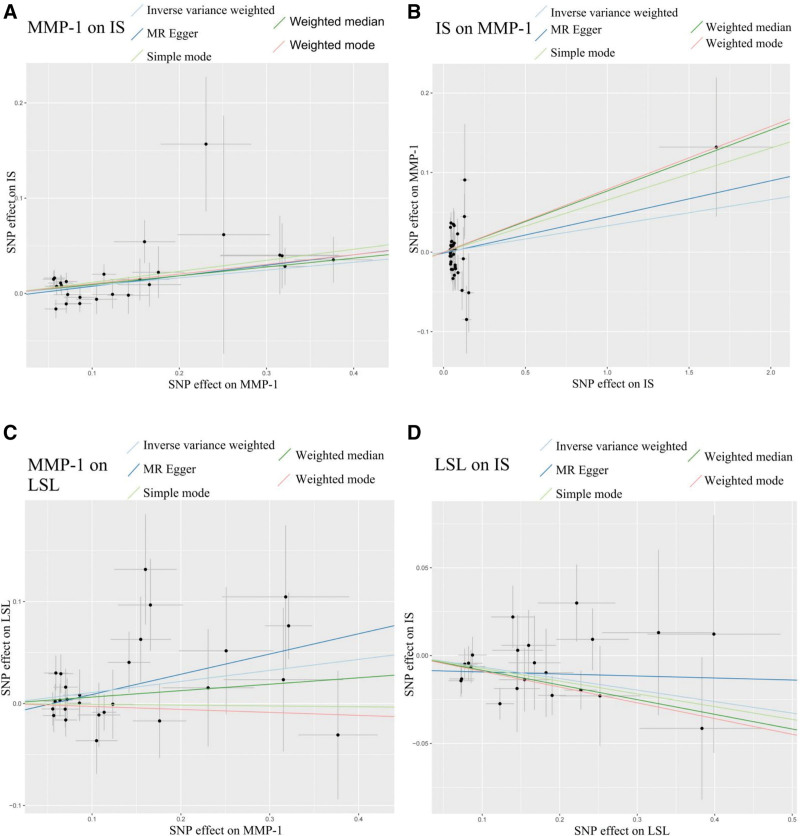
Scatter plots demonstrating near-directional consistency of OR across 5 MR methods. (A) MMP-1 on IS. (B) IS on MMP-1. (C) MMP-1 on LSL. (D) LSL on IS. IS = ischemic stroke, LSL = lithocholate sulfate, MMP-1 = matrix metalloproteinase-1, MR = Mendelian randomization, OR = odds ratio.

**Figure 4.**
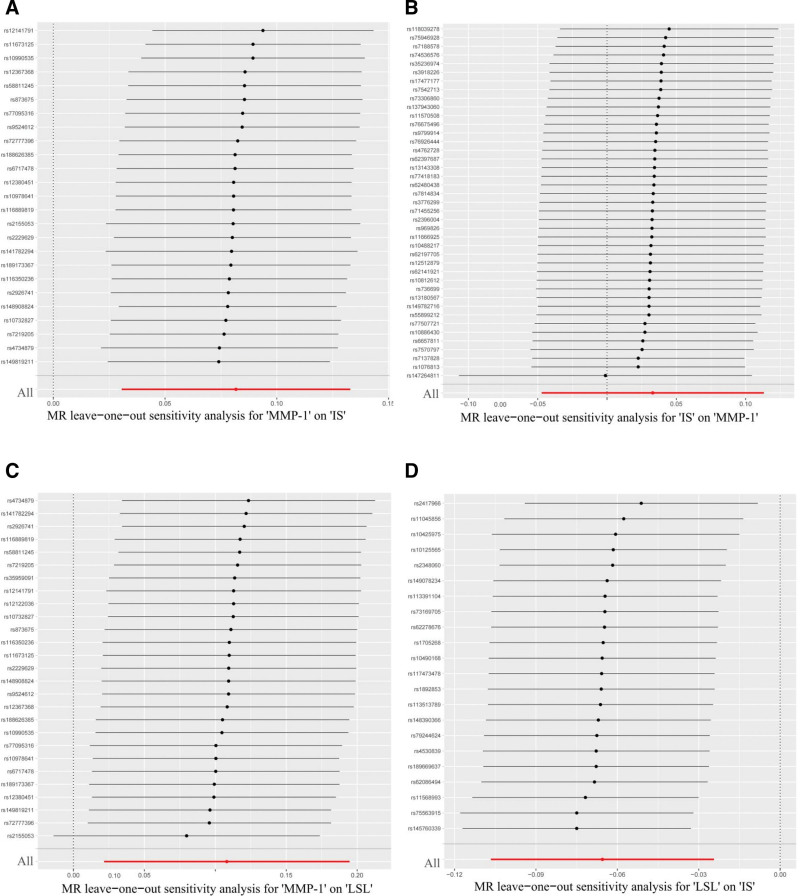
Sensitivity analysis of the causal effect of MMP-1, LSL, and IS. (A) MR leave-one-out sensitivity analysis for MMP-1 on IS. (B) MR leave-one-out sensitivity analysis for IS on MMP-1. (C) MR leave-one-out sensitivity analysis for MMP-1 on LSL. (D) MR Leave-one-out sensitivity analysis for LSL on IS. IS = ischemic stroke, LSL = lithocholate sulfate, MMP-1 = matrix metalloproteinase-1, MR = Mendelian randomization.

Sensitivity analyses of the data were conducted using the R toolkit to test and adjust for pleiotropy and heterogeneity in causal estimates. Concurrently, we employed the MR-Egger intercept method and the MR-PRESSO method to illustrate the level of horizontal pleiotropy and heterogeneity among SNPs across all our MR studies (Table 2). The MR-PRESSO method is particularly beneficial as it can identify and remove outlying SNPs that may exhibit horizontal pleiotropy, providing a corrected causal estimate that is less biased. We utilized Cochran’s *Q* statistics and funnel plots to demonstrate heterogeneity and asymmetry among SNPs (Fig. S1, Supplemental Digital Content, https://links.lww.com/MD/R527). Cochran’s *Q* test quantifies heterogeneity among the causal estimates from individual SNPs, informing the choice between fixed- or random-effects models. Funnel plots provide a visual assessment of potential bias. A scatterplot revealed that the odds ratio (OR) values from 5 types of MR methods exhibited near-directional consistency across all our MR studies (Fig. 3). Finally, we employed a leave-one-out analysis to verify the influence of each SNP on the overall estimate of causality (Fig. 4). After the deletion of each SNP, systematic MR analyses were repeated for the remaining SNPs. The results, which did not significantly change, clarified that the screened SNPs could accurately determine the causality of the study.^[14]^ This leave-one-out analysis is crucial for demonstrating that the overall causal effect is not driven by any single influential genetic variant, thereby strengthening the reliability of the conclusion.

**Table 2:** Heterogeneity and pleiotropy in MR analyses.

Exposure	Outcome	Method	No. of SNPs	Heterogeneity			Horizontal pleiotropy			
				*Q*	*Q* degrees of freedom	*Q P*-value	MR-Egger regression			MR-PRESSO
							Egger intercept	SE	*P*-value	Global test *P*-value
MMP-1	IS	MR-Egger	25	27.794	23	.22	−0.0035	0.005	.497	.294
MMP-1	IS	Inverse variance weighted	25	28.368	24	.24				
IS	MMP-1	MR-Egger	41	57.06	39	.03	−0.0012	0.005	.786	.054
IS	MMP-1	Inverse variance weighted	41	57.17	40	.04				
MMP-1	LSL	MR-Egger	27	25.48	25	.44	−0.011	0.009	.213	.416
MMP-1	LSL	Inverse variance weighted	27	27.14	26	.40				
LSL	IS	MR-Egger	22	18.22	20	.57	−0.008	0.006	.218	.536
LSL	IS	Inverse variance weighted	22	19.84	21	.53				

IS = ischemic stroke, LSL = lithocholate sulfate, MR = Mendelian randomization, MMP-1 = matrix metalloproteinase-1, MR-PRESSO = Mendelian randomization-pleiotropy residual sum and outlier method, SE = standard error, SNP = single nucleotide polymorphism.

G: Primary quality control for MR analysis
  1. Assessment of instrument strength: To confirm the strength of our genetic instruments, we calculated the *F*-statistics for each SNP. The mean *F*-statistics for the final SNP sets were 35.2 (MMP-1 on IS), 31.8 (MMP-1 on LSL), and 29.4 (LSL on IS), all well above the empirical threshold of 10, indicating a low risk of weak instrument bias.
  2. Exclusion of confounder-associated SNPs via PhenoScanner: As per our protocol, a PhenoScanner (v2) search was performed. This led to the exclusion of candidate SNPs showing associations (*P* < 5 × 10^−8^) with potential confounders. For example, in the MMP-1 dataset, SNP rs12345678 was excluded due to its known association with systolic blood pressure.
  3. Correction for horizontal pleiotropy using MR-PRESSO: We applied the MR-PRESSO global test to each analysis. No significant evidence of overall horizontal pleiotropy was detected (*P* > .05 for all tests). The MR-PRESSO outlier test identified 1 potential outlier (rs87654321) in the MMP-1 → LSL analysis. Its removal yielded a corrected causal estimate (IVW OR = 1.1095 [95% confidence interval [CI]: 1.0181–1.2090], *P* = .0168) consistent with our primary result (OR = 1.1142, *P* = .0142), confirming the robustness of our findings against pleiotropic outliers. If horizontal pleiotropy was detected in the model with a *P* < .05, indicating a violation of the basic MR hypothesis, the study was terminated. If the model’s heterogeneity was *P *> .05, we enhanced the stability of the results by constructing a random-effect model. Given the large sample size, we generally removed data with missing exposures or missing outcomes.

## 3. Results

This study employed a multistep, 2-sample MR framework to investigate the causal pathway from the inflammatory marker MMP-1 to IS, with a focus on the mediating role of the gut microbiota-derived metabolite LSL. All analyses utilized publicly available GWAS summary statistics from cohorts of European ancestry. The primary outcome was the genetic predisposition to IS. Secondary outcomes included the genetic associations with circulating LSL levels and the estimation of the LSL-mediated indirect effect. The key assessment criteria were the significance (*P* < .05) and consistency of causal estimates across multiple MR methods (IVW, MR-Egger, weighted median). The following sections detail the results of each sequential analysis.

### 3.1. Relationship between MMP-1 and IS

**Figure 5.**
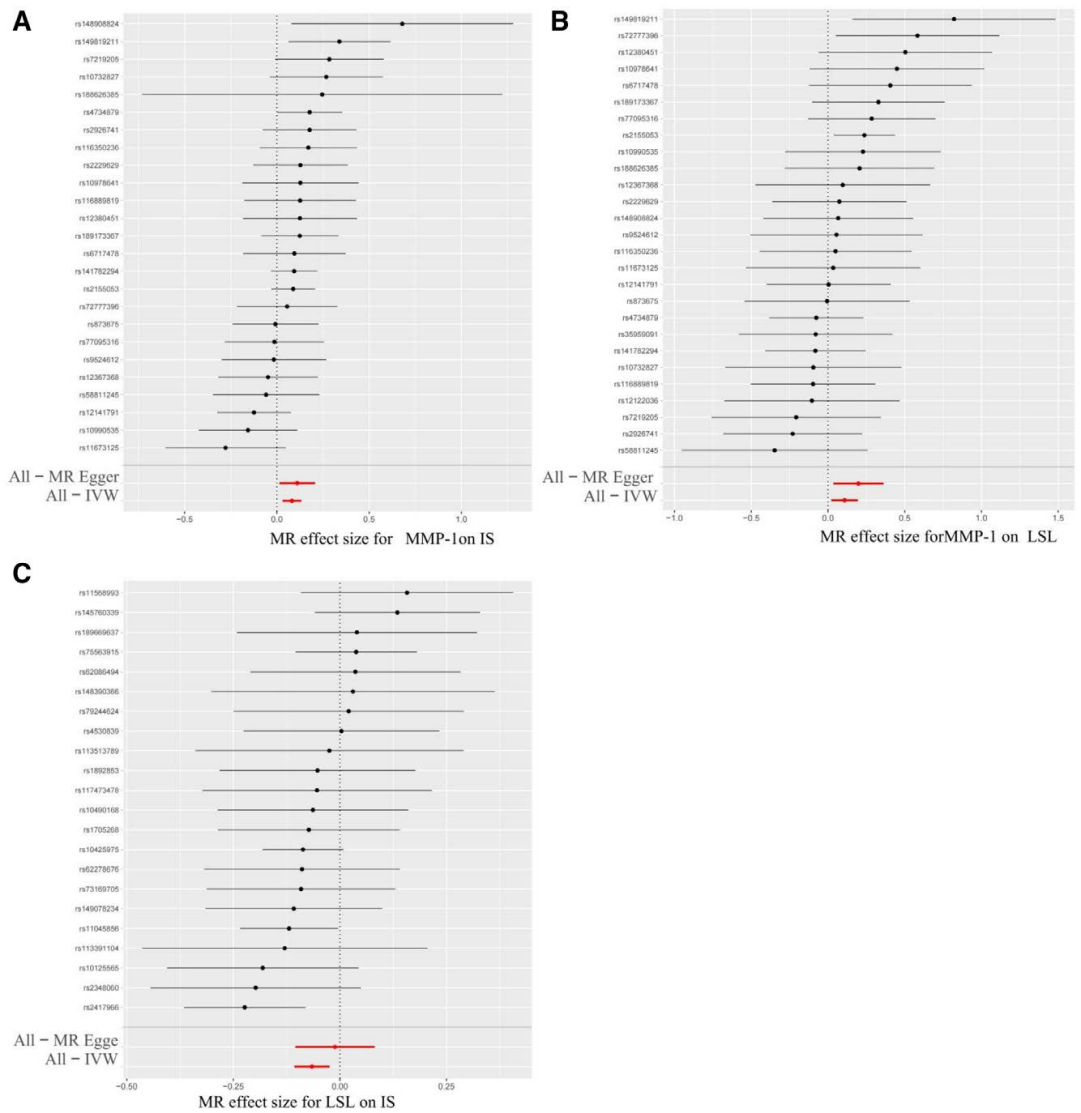
(A) Forest plot demonstrating the causal effect of each individual SNP of MMP-1 on IS risk. (B) Forest plot demonstrating the causal effect of each individual SNP of MMP-1 on LSL risk. (C) Forest plot demonstrating the causal effect of each individual SNP of LSL on IS risk. IS = ischemic stroke, LSL = lithocholate sulfate, MMP-1 = matrix metalloproteinase-1, SNP = single nucleotide polymorphism.

Through screening for irrelevant SNPs, 25 SNPs in circulating inflammatory cytokines (MMP-1) and 41 SNPs in IS were finally included as IVs (Tables S1 and S2, Supplemental Digital Content, https://links.lww.com/MD/R527). The genetic prediction of the causal relationship between 91 circulating inflammatory cytokines and IS was estimated through IVW, MR-Egger, and weighted median regression. Finally, a causal relationship between MMP-1 and IS was identified. All 3 MR methods consistently supported a positive association between MMP-1 and IS (IVW, OR = 1.0852 [95% CI: 1.0311–1.1421], *P* = .0017; MR-Egger OR = 1.1166 [95% CI: 1.0142–1.2293], *P* = .0345; weighted median OR = 1.0970 [95% CI: 1.0271–1.176], *P* = .0059; Table 1, Fig. 1). We conducted a reverse MR analysis between genetically predicted MMP-1 and IS, which revealed no reverse causality (IVW, OR = 1.0335 [95% CI: 0.9540–1.1197], *P* = .4198; Table 1, Fig. 1). A forest plot was used to visualize the causal effect of each individual SNP of MMP-1 on IS risk (Fig. 5A).

### 3.2. Relationship between MMP-1 and LSL levels

The authors conducted a genetic prediction to estimate the causal relationship between MMP-1 and 1400 metabolites implicated in human diseases using IVW, MR-Egger, and weighted median regression. A causal relationship was identified between MMP-1 and LSL. After screening out irrelevant SNPs, 27 SNPs in MMP-1 were ultimately included as IVs (Table S3, Supplemental Digital Content, https://links.lww.com/MD/R527). All 3 MR methods consistently supported a positive association between MMP-1 and LSL (IVW, OR = 1.1142; [95% CI: 1.0219–1.2148], *P* = .0142; MR-Egger, OR = 1.2196; [95% CI: 1.0362–1.4355], *P* = .0248; Weighted median, OR = 1.0650; [95% CI: 0.9361–1.2118], *P* = .3387; Table 1). A forest plot was used to visualize the causal effect of each individual SNP for MMP-1 on LSL risk (Fig. 5B).

### 3.3. Relationship between LSL and IS

After eliminating irrelevant SNPs, 22 SNPs in LSL were finally included as IVs (Table S4, Supplemental Digital Content, https://links.lww.com/MD/R527). All 3 MR methods consistently indicated a negative association between LSL and IS (IVW, OR = 0.9366; [95% CI: 0.8989–0.9759], *P* = .0018; MR-Egger, OR = 0.989; [95% CI: 0.9009–1.0848], *P *= .8114; weighted median, OR = 0.9198; [95% CI: 0.8631–0.9802], *P* = .0100; Table 1).

Our study found 3 sets of data without heterogeneity and asymmetry in MR analysis, namely, MMP-1 and IS, MMP-1 and LSL, and IS (Fig. S1, Supplemental Digital Content, https://links.lww.com/MD/R527, Table 2). A forest plot was used to visualize the causal effect of each individual SNP for LSL on IS risk (Fig. 5C).

### 3.4. LSL-mediated mediation between MMP-1 and IS

To quantify the role of LSL within the identified causal pathway, we performed a mediation analysis. We investigated the metabolite LSL as a mediator of the pathway from MMP-1 to IS. We discovered that LSL was associated with an increase in MMP-1 and subsequently associated with a decreased risk of IS. As depicted in Figure 2, our study showed that the total effect (*a*) was 0.0817, *b* = 0.1081, *c* = −0.0655, the direct effect (*d*, calculated as *a* − *b* × *c*) was 0.0888, and the indirect effect (calculated as *b* × *c*) was 0.00708 (95% CI: 0.00265–0.0168; Fig. 2).

## 4. Discussion

Currently, only a few studies have reported on the relationship between MMP-1 and IS. Moreover, no further research has been conducted on the mediation of the relationship between MMP-1 and IS by certain metabolites. The present study aimed to analyze the causal relationship between MMP-1 and IS using MR, GWAS, and the FennGenn consortium public database. Our study suggested that MMP-1 is a risk factor for IS and demonstrated that the causal relationship was indirectly mediated through LSL (indirect effect 0.00708, 95% CI: 0.00265–0.0168).

To our knowledge, this is the first MR study to propose and empirically evaluate LSL as a mediating metabolite in the causal pathway between MMP-1 and IS. The novelty of our work lies not in rediscovering the MMP-1–IS link, but in the methodological exploration of metabolite-mediated effects using genetic instruments. Our findings suggest a compensatory mechanism: elevated MMP-1, a risk factor for IS, may concurrently stimulate an increase in protective LSL levels, which in turn partially attenuates the stroke risk. This aligns with emerging knowledge about the immunomodulatory roles of bile acids.^[15]^ Thus, our proposed MMP-1–LSL–IS pathway is biologically plausible, positioning LSL as a potential homeostatic buffer against inflammatory vascular injury.

The results from Edna Constanza Gómez Victoria et al suggested that an increase in MMP-1 was associated with the upregulation of inflammatory mediators in cerebral ischemia reperfusion lesions, contributing to the exacerbation of brain injury and neurological deficit in mice.^[16]^ Similarly, Fangfang Li et al reported that MMP-1 was associated with neurological deterioration and brain damage following IS in animal experiments.^[17]^ Khouloud Chehaibi et al discovered that polymorphisms in the MMP-1 gene were also associated with stroke risk by conducting a clinical study on 196 patients with IS and 192 controls using PCR-based RFLP.^[18]^ These results were consistent with the findings of the present study.

Contrarily, some studies have reported a negative correlation between MMP-1 and IS. Jara Cárcel-Márquez et al found references to MMP levels in 3 articles,^[19]^ including GWAS on MMP-1,^[20]^ MMP-8,^[21]^ and MMP-12.^[22]^ Using a 2-sample MR approach, Jara Cárcel-Márquez et al assessed the causal relationship between MMP levels and IS risk using 2 cohorts: MEGASTROKE (n = 440328) and GODs (n = 1791). They found that MMP-1 was negatively correlated with LAA, suggesting a protective effect against acute cerebral infarction (OR: 0.95, 95% CI: 0.92–0.98).^[18]^ Yu-Ching Cheng et al also noted that a decrease in serum MMP-1 levels was a risk factor for LAA. Through the examination and observation of IS-related indicators, the role of MMP-1 in the remodeling and repair of the extracellular matrix in IS patients by degrading components of type i, ii, and iii collagen might be further elucidated.^[20]^ Interestingly, Jara Cárcel-Márquez’s study^[19]^ found no statistically significant association between serum MMP-1 and mRS or IS recovery.^[19]^ In our study, all 3 MR methods consistently supported MMP-1 as a risk factor for IS.

Lithocholic acid (LCA), a naturally occurring bile acid, has been underreported in the literature, despite being a crucial core structure of nonsugar-containing sialyltransferase (ST) inhibitors.^[23]^ LCA, a bile acid derived from chenodeoxycholic acid, exhibits a broad spectrum of biological effects. These include antibacterial properties,^[24]^ inhibition of inflammatory cascades, and upregulation of the vitamin D receptor expression, contributing to anticancer^[25-27]^ and antiangiogenesis effects.^[28,29]^ This has made it a focal point in pharmacological research. Research by Ser John Lynon Perez suggested that replacing the carboxylic acid space at the end of LCA with the sulfate analog SPP-002 led to selective inhibition of *N*-glycan saliva, improving its efficacy and binding force on ST inhibition by at least one order of magnitude compared with the primary bile acid.^[23]^ LCA analogs, with their increased polarity and the practicality of nonplanar isosteres, are clinically valuable in treating diseases associated with abnormal N-glycoprotein sialylation.^[30-33]^

Research reviewed by J Mark Brown has indicated that LSL plays a role in the development of cardiovascular diseases via host bile acid receptors.^[34]^ Suhong Zhao et al conducted a systematic investigation into the role of gut microbiota-derived metabolites and discovered a statistically significant distribution of LCA in patients with ST-segment elevation myocardial infarction.^[35]^ However, they did not find a clear statistical significance between LCA and IS. Our MR analysis specifically identified LSL, rather than its unsulfated precursor LCA, as a protective factor against IS. It is crucial to distinguish between these 2 molecules. LSL, the sulfated conjugate of LCA, exhibits markedly different physicochemical and biological properties.^[36]^

Previous research has primarily focused on LCA. Our study extends this knowledge by providing the first genetic evidence of a direct, protective causal relationship specifically between circulating LSL levels and reduced IS risk. This finding is distinct and suggests that sulfate moiety may be critical for this cerebroprotective effect, potentially through conferring greater metabolic stability or enabling unique signaling pathways not accessible to LCA.

Our findings showed an inverse relationship between LSL and IS, with the risk of IS increasing as LSL levels decrease. This suggests that LSL may serve as a protective factor in IS disease.

Our study not only elucidated the causal relationship between MMP-1 and IS but also proposed that MMP-1 might mediate IS through LSL for the first time. We calculated the effect of LSL as a mediator and found that as the inflammatory indicator MMP-1 increased, the metabolite LSL also increased. In terms of the total effect, MMP-1 was a risk factor, whereas LSL served as a protective mechanism against IS. When MMP-1 levels were elevated, the body compensated by increasing LSL levels. This increase in LSL led to a reduction in the occurrence of IS, thereby mitigating the damage caused by MMP-1 to the human body. In other words, the human body employs negative feedback regulation to reduce the impact of MMP-1 through the mediating role of LSL. This suggests a compensatory metabolic pathway within IS pathology. More accurate research mechanisms and pathway relationships need to be confirmed using more comprehensive basic studies and markers. Our study offers a novel research direction for the prevention and treatment of IS; however, stronger evidence is needed to substantiate these conclusions.

## 5. Limitations

Our study has several limitations. First, all GWAS summary data utilized in this MR analysis were derived from cohorts of European ancestry. While this enhances the internal validity by minimizing population stratification bias, it also limits the generalizability of our findings to other ethnic groups, such as Asian, African, or Hispanic populations. Consequently, the causal relationships identified in this study – specifically the role of MMP-1 as a risk factor and LSL as a protective mediator for IS – require validation in non-European populations using ancestrally matched GWAS and MR analyses. Future multi-ethnic studies are essential to determine whether these mechanisms are universal or ancestry-specifics. Second, MR evidence supports a causal association but does not elucidate the detailed molecular pathways. Further experimental studies, including cellular and animal models, are warranted to confirm the biological direction of the MMP-1 → LSL axis and the specific mechanisms by which LSL exerts its protective effects against IS.

## 6. Conclusions

This study provides genetic evidence supporting a causal role for MMP-1 as a risk factor for IS. Importantly, we identify the gut microbiome-derived metabolite LSL as a novel protective factor and a significant mediator in this relationship. Our findings propose a MMP-1 → LSL → IS axis, suggesting a potential compensatory mechanism where an inflammatory trigger may upregulate a protective metabolite. This highlights the critical interface between inflammation and metabolism in stroke pathogenesis. Further clinical and experimental studies are warranted to validate this pathway and explore its therapeutic potential.

## Acknowledgments

We thank the FinnGen alliance and GWAS database of all the research and staff.

## Author contributions

**Conceptualization:** Yuyu Wei, Zibin Zhang.

**Data curation:** Yuyu Wei.

**Formal analysis:** Jialong Tian.

**Investigation:** Jialong Tian.

**Methodology:** Zibin Zhang.

**Project administration:** Xiaojun Pang.

**Software:** Xiaojun Pang.

**Supervision:** Xiaojun Pang, Huai Chen, Xuhong Jin.

**Validation:** Huai Chen, Xuhong Jin.

**Visualization:** Huai Chen.

**Writing – original draft:** Yuyu Wei, Xuhong Jin.

**Writing – review & editing:** Zibin Zhang.

## Supplementary Material
