# Supplementary material for: Mendelian randomization study of lithocholate sulfate mediating the effect of MMP-1 on ischemic stroke
Source: Medicine (Baltimore). 2026 Mar 13;105(11):e48010. doi: 10.1097/MD.0000000000048010 (PMC12991698; doi:10.1097/MD.0000000000048010)
Supplement: Supplementary file 1 [file medi-105-e48010-s001.pdf]

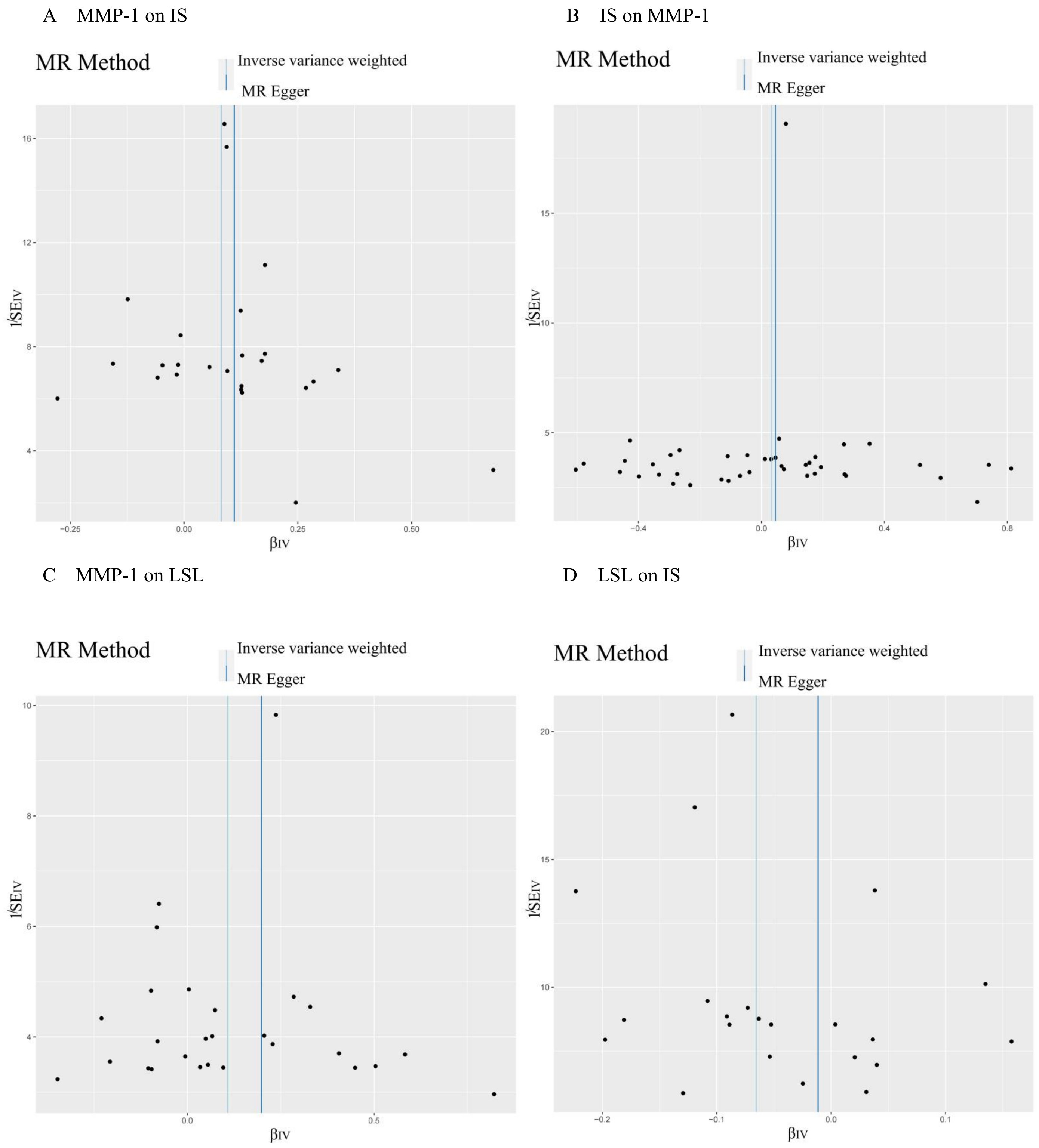

Figure S1: Funnel plots illustrating heterogeneity among SNPs in four sets of data (A, B, C, and D).

Supplementary Table S1: Characteristics of significant SNPs with genome-wide associations (P<5×10-5) for MMP-1 on IS

SNP	effect_al	other_all	effect_al	other_all	beta.expo	beta.outc	eaf.expos	eaf.outco	remove	palindrom	ambiguous	id.outcom	pos.outco	pval.outc	se.outcom	outcome
rs1073282	A	G	A	G	0.0558	0.014947	0.3498	0.372556	FALSE	FALSE	FALSE	1aSZ1N	84132151	0.085743	0.008699	outcome
rs1097864	A	T	A	T	0.0649	0.008265	0.7462	0.791325	FALSE	TRUE	FALSE	1aSZ1N	1.07E+08	0.427188	0.01041	outcome
rs1099053	T	C	T	C	0.0703	-0.011	0.253	0.26549	FALSE	FALSE	FALSE	1aSZ1N	96328727	0.250628	0.009576	outcome
rs1163502	A	T	A	T	0.0639	0.010896	0.3845	0.4122	FALSE	TRUE	FALSE	1aSZ1N	1.24E+08	0.203859	0.008575	outcome
rs1167312	T	C	T	C	-0.0585	0.016276	0.296	0.247362	FALSE	FALSE	FALSE	1aSZ1N	14652936	0.094632	0.009738	outcome
rs1168898	A	G	A	G	-0.176	-0.0222	0.0455	0.024986	FALSE	FALSE	FALSE	1aSZ1N	1.03E+08	0.412973	0.02712	outcome
rs1214179	A	G	A	G	0.0859	-0.01064	0.6981	0.63455	FALSE	FALSE	FALSE	1aSZ1N	1.56E+08	0.223758	0.008745	outcome
rs1236736	T	G	T	G	0.0861	-0.00411	0.1146	0.14967	FALSE	FALSE	FALSE	1aSZ1N	83200264	0.728286	0.01182	outcome
rs1238045	T	C	T	C	-0.0593	-0.00742	0.7098	0.715395	FALSE	FALSE	FALSE	1aSZ1N	9191014	0.426576	0.009337	outcome
rs1417822	A	G	A	G	0.3767	0.035289	0.9777	0.967804	FALSE	FALSE	FALSE	1aSZ1N	1.02E+08	0.14199	0.024032	outcome
rs1489088	A	G	A	G	-0.2306	-0.15673	0.0214	0.003783	FALSE	FALSE	FALSE	1aSZ1N	24654031	0.026537	0.070655	outcome
rs1498192	A	G	A	G	-0.1601	-0.05425	0.0417	0.035984	FALSE	FALSE	FALSE	1aSZ1N	21009900	0.016099	0.022543	outcome
rs1886263	T	C	T	C	-0.2509	-0.06167	0.0177	0.00122	FALSE	FALSE	FALSE	1aSZ1N	1.21E+08	0.621621	0.124944	outcome
rs1891733	T	C	T	C	0.3179	0.039487	0.0131	0.017033	FALSE	FALSE	FALSE	1aSZ1N	8720275	0.243865	0.033883	outcome
rs2155053	T	C	T	C	-0.3212	-0.02841	0.9461	0.950291	FALSE	FALSE	FALSE	1aSZ1N	1.03E+08	0.143053	0.019399	outcome
rs2229629	A	G	A	G	-0.3153	-0.04022	0.0135	0.010701	FALSE	FALSE	FALSE	1aSZ1N	74889400	0.32809	0.041129	outcome
rs2926741	T	C	T	C	-0.0704	-0.01251	0.3436	0.31032	FALSE	FALSE	FALSE	1aSZ1N	56704813	0.169912	0.009111	outcome
rs4734879	A	G	A	G	0.1136	0.020204	0.7422	0.778074	FALSE	FALSE	FALSE	1aSZ1N	1.06E+08	0.047596	0.010199	outcome
rs484915	A	T	A	T	-0.3517	0.012973	0.5613	0.635854	FALSE	TRUE	TRUE	1aSZ1N	1.03E+08	0.13938	0.008777	outcome
rs5881124	T	C	T	C	-0.1051	0.006131	0.9266	0.919262	FALSE	FALSE	FALSE	1aSZ1N	62157046	0.691154	0.015431	outcome
rs6717478	A	T	A	T	0.1548	0.014704	0.966	0.961818	FALSE	TRUE	FALSE	1aSZ1N	63429919	0.502195	0.021912	outcome
rs7219205	A	G	A	G	0.0569	0.016183	0.5023	0.579277	FALSE	FALSE	FALSE	1aSZ1N	4448017	0.058196	0.008543	outcome
rs7277739	A	G	A	G	0.1657	0.009237	0.9671	0.965268	FALSE	FALSE	FALSE	1aSZ1N	10782980	0.687523	0.022965	outcome
rs7709531	T	G	T	G	-0.1416	0.001857	0.0705	0.049771	FALSE	FALSE	FALSE	1aSZ1N	1.03E+08	0.923712	0.019388	outcome
rs873675	T	C	T	C	0.1234	-0.00096	0.0684	0.091462	FALSE	FALSE	FALSE	1aSZ1N	65005770	0.947716	0.01463	outcome
rs9524612	T	C	T	C	-0.072	0.001166	0.177	0.209831	FALSE	FALSE	FALSE	1aSZ1N	94777638	0.910662	0.010392	outcome

SNP	mr_keep.o	pval_orig	data_sour	se.exposu	pval.expo	samplesiz	exposure	mr_keep.e	pval_orig	id.exposu	data_sour	action	SNP_index	mr_keep	samplesiz	R2	f	meanf
rs1073282	TRUE	reported	textfile	0.0124	6.80E-06	14742	exposure	TRUE	reported	VheQ7B	textfile	2	1	TRUE	NA	0.001372	20.24725	65.17072
rs1097864	TRUE	reported	textfile	0.014	3.56E-06	14296	exposure	TRUE	reported	VheQ7B	textfile	2	1	TRUE	NA	0.001501	21.48684	65.17072
rs1099053	TRUE	reported	textfile	0.0135	1.91E-07	14734	exposure	TRUE	reported	VheQ7B	textfile	2	1	TRUE	NA	0.001837	27.11341	65.17072
rs1163502	TRUE	reported	textfile	0.0142	6.80E-06	10717	exposure	TRUE	reported	VheQ7B	textfile	2	1	TRUE	NA	0.001886	20.24622	65.17072
rs1167312	TRUE	reported	textfile	0.0127	4.10E-06	14741	exposure	TRUE	reported	VheQ7B	textfile	2	1	TRUE	NA	0.001437	21.21511	65.17072
rs1168898	TRUE	reported	textfile	0.03	4.45E-09	14744	exposure	TRUE	reported	VheQ7B	textfile	2	1	TRUE	NA	0.002329	34.41311	65.17072
rs1214179	TRUE	reported	textfile	0.013	3.90E-11	14296	exposure	TRUE	reported	VheQ7B	textfile	2	1	TRUE	NA	0.003045	43.65549	65.17072
rs1236736	TRUE	reported	textfile	0.0186	3.67E-06	14744	exposure	TRUE	reported	VheQ7B	textfile	2	1	TRUE	NA	0.001451	21.42503	65.17072
rs1238045	TRUE	reported	textfile	0.0133	8.25E-06	14296	exposure	TRUE	reported	VheQ7B	textfile	2	1	TRUE	NA	0.001389	19.87675	65.17072
rs1417822	TRUE	reported	textfile	0.0445	2.56E-17	14731	exposure	TRUE	reported	VheQ7B	textfile	2	1	TRUE	NA	0.004841	71.64935	65.17072
rs1489088	TRUE	reported	textfile	0.0517	8.18E-06	12935	exposure	TRUE	reported	VheQ7B	textfile	2	1	TRUE	NA	0.001536	19.89163	65.17072
rs1498192	TRUE	reported	textfile	0.0351	5.08E-06	14744	exposure	TRUE	reported	VheQ7B	textfile	2	1	TRUE	NA	0.001409	20.80221	65.17072
rs1886263	TRUE	reported	textfile	0.053	2.20E-06	12935	exposure	TRUE	reported	VheQ7B	textfile	2	1	TRUE	NA	0.00173	22.40693	65.17072
rs1891733	TRUE	reported	textfile	0.0714	8.49E-06	11925	exposure	TRUE	reported	VheQ7B	textfile	2	1	TRUE	NA	0.00166	19.82037	65.17072
rs2155053	TRUE	reported	textfile	0.0264	4.68E-34	14741	exposure	TRUE	reported	VheQ7B	textfile	2	1	TRUE	NA	0.009942	148.0077	65.17072
rs2229629	TRUE	reported	textfile	0.0673	2.80E-06	10887	exposure	TRUE	reported	VheQ7B	textfile	2	1	TRUE	NA	0.002012	21.94512	65.17072
rs2926741	TRUE	reported	textfile	0.0126	2.31E-08	14296	exposure	TRUE	reported	VheQ7B	textfile	2	1	TRUE	NA	0.002179	31.21357	65.17072
rs4734879	TRUE	reported	textfile	0.0137	1.11E-16	14296	exposure	TRUE	reported	VheQ7B	textfile	2	1	TRUE	NA	0.004786	68.74716	65.17072
rs484915	TRUE	reported	textfile	0.0119	########	14295	exposure	TRUE	reported	VheQ7B	textfile	2	1	FALSE	NA	0.057585	873.3535	65.17072
rs5881124	TRUE	reported	textfile	0.0234	7.07E-06	14723	exposure	TRUE	reported	VheQ7B	textfile	2	1	TRUE	NA	0.001368	20.17041	65.17072
rs6717478	TRUE	reported	textfile	0.0338	4.65E-06	14742	exposure	TRUE	reported	VheQ7B	textfile	2	1	TRUE	NA	0.001421	20.97247	65.17072
rs7219205	TRUE	reported	textfile	0.0121	2.57E-06	14296	exposure	TRUE	reported	VheQ7B	textfile	2	1	TRUE	NA	0.001544	22.11022	65.17072
rs7277739	TRUE	reported	textfile	0.0364	5.31E-06	14744	exposure	TRUE	reported	VheQ7B	textfile	2	1	TRUE	NA	0.001404	20.71969	65.17072
rs7709531	TRUE	reported	textfile	0.0238	2.69E-09	14744	exposure	TRUE	reported	VheQ7B	textfile	2	1	TRUE	NA	0.002395	35.3927	65.17072
rs873675	TRUE	reported	textfile	0.0234	1.34E-07	14742	exposure	TRUE	reported	VheQ7B	textfile	2	1	TRUE	NA	0.001883	27.80607	65.17072
rs9524612	TRUE	reported	textfile	0.0162	8.81E-06	14296	exposure	TRUE	reported	VheQ7B	textfile	2	1	TRUE	NA	0.00138	19.75032	65.17072

Supplementary Table S2: Characteristics of significant SNPs with genome-wide associations (P<5×10-5) for IS on MMP-1

SNP	effect_al	other_all	effect_al	other_all	beta.expo	beta.outc	eaf.expos	eaf.outco	remove	palindrom	ambiguous	id.outcom	se.outcom	pval.outc	samplesiz	outcome	mr_keep.o	pval_orig	data_sour	pos.expos	pval.expo	se.exposu	exposure	mr_keep.e	pval_orig	id.exposu	data_sour	samplesiz	action	SNP_index	mr_keep	R2	f	meanf
rs1048821	T	A	T	A	0.047017	0.007	0.208999	0.1846	FALSE	TRUE	FALSE	EmctNB	0.0155	0.652	14296	outcome	TRUE	reported	textfile	1.33E+08	4.92E-06	0.010292	exposure	TRUE	reported	uIS7w6	textfile	365318	2	1	TRUE	5.71E-05	20.86997	28.95116
rs1076813	G	A	G	A	0.042048	0.0311	0.506161	0.5083	FALSE	FALSE	FALSE	EmctNB	0.0119	0.00896	14296	outcome	TRUE	reported	textfile	1.04E+08	6.03E-07	0.008426	exposure	TRUE	reported	uIS7w6	textfile	365318	2	1	TRUE	6.82E-05	24.90205	28.95116
rs1081261	C	T	C	T	-0.04389	-0.0085	0.303109	0.3015	FALSE	FALSE	FALSE	EmctNB	0.0128	0.507	14733	outcome	TRUE	reported	textfile	27545547	1.77E-06	0.009185	exposure	TRUE	reported	uIS7w6	textfile	365318	2	1	TRUE	6.25E-05	22.82989	28.95116
rs1088643	G	A	G	A	0.085321	0.0229	0.093131	0.1255	FALSE	FALSE	FALSE	EmctNB	0.0191	0.231	14296	outcome	TRUE	reported	textfile	1.19E+08	4.43E-09	0.014542	exposure	TRUE	reported	uIS7w6	textfile	365318	2	1	TRUE	9.42E-05	34.42414	28.95116
rs1157050	A	C	A	C	0.044865	-0.0123	0.243779	0.2221	FALSE	FALSE	FALSE	EmctNB	0.0144	0.393	14296	outcome	TRUE	reported	textfile	47151194	4.62E-06	0.009793	exposure	TRUE	reported	uIS7w6	textfile	365318	2	1	TRUE	5.75E-05	20.99038	28.95116
rs1166692	G	A	G	A	-0.0609	-0.0035	0.2298	0.2869	FALSE	FALSE	FALSE	EmctNB	0.0129	0.786	14742	outcome	TRUE	reported	textfile	11065175	1.32E-09	0.010041	exposure	TRUE	reported	uIS7w6	textfile	365318	2	1	TRUE	0.000101	36.78903	28.95116
rs1180392	A	G	A	G	0.112692	-0.0482	0.045662	0.0669	FALSE	FALSE	FALSE	EmctNB	0.0243	0.0473	14725	outcome	TRUE	reported	textfile	1.61E+08	1.66E-08	0.019965	exposure	TRUE	reported	uIS7w6	textfile	365318	2	1	TRUE	8.72E-05	31.859	28.95116
rs1251287	T	G	T	G	0.056853	0.0082	0.229141	0.1644	FALSE	FALSE	FALSE	EmctNB	0.0161	0.611	14739	outcome	TRUE	reported	textfile	1.55E+08	1.20E-08	0.009975	exposure	TRUE	reported	uIS7w6	textfile	365318	2	1	TRUE	8.89E-05	32.48434	28.95116
rs1314330	G	T	G	T	-0.04568	0.0049	0.693099	0.7911	FALSE	FALSE	FALSE	EmctNB	0.0163	0.764	11793	outcome	TRUE	reported	textfile	1.11E+08	5.29E-07	0.009108	exposure	TRUE	reported	uIS7w6	textfile	365318	2	1	TRUE	6.89E-05	25.15651	28.95116
rs1318056	T	C	T	C	-0.048	-0.0132	0.225508	0.1748	FALSE	FALSE	FALSE	EmctNB	0.0158	0.403	14296	outcome	TRUE	reported	textfile	1.59E+08	2.17E-06	0.010134	exposure	TRUE	reported	uIS7w6	textfile	365318	2	1	TRUE	6.14E-05	22.4357	28.95116
rs1379430	A	G	A	G	0.15336	-0.0511	0.018109	0.0234	FALSE	FALSE	FALSE	EmctNB	0.0497	0.304	13394	outcome	TRUE	reported	textfile	99613860	1.09E-06	0.031459	exposure	TRUE	reported	uIS7w6	textfile	365318	2	1	TRUE	6.50E-05	23.76468	28.95116
rs1472648	G	C	G	C	-1.66696	-0.132	0.000292	0.0069	FALSE	TRUE	FALSE	EmctNB	0.0874	0.131	11309	outcome	TRUE	reported	textfile	89660229	1.88E-06	0.349746	exposure	TRUE	reported	uIS7w6	textfile	365318	2	1	TRUE	6.22E-05	22.71655	28.95116
rs1497827	G	A	G	A	0.129175	0.0907	0.022142	0.011	FALSE	FALSE	FALSE	EmctNB	0.0701	0.196	13394	outcome	TRUE	reported	textfile	84108925	3.72E-06	0.027922	exposure	TRUE	reported	uIS7w6	textfile	365318	2	1	TRUE	5.86E-05	21.40205	28.95116
rs1747717	C	T	C	T	0.047129	-0.0217	0.296346	0.2002	FALSE	FALSE	FALSE	EmctNB	0.0147	0.14	14744	outcome	TRUE	reported	textfile	1.07E+08	2.94E-07	0.009191	exposure	TRUE	reported	uIS7w6	textfile	365318	2	1	TRUE	7.20E-05	26.29169	28.95116
rs2396004	G	A	G	A	-0.04141	-0.0027	0.620566	0.5641	FALSE	FALSE	FALSE	EmctNB	0.0119	0.821	14729	outcome	TRUE	reported	textfile	43388113	1.65E-06	0.00864	exposure	TRUE	reported	uIS7w6	textfile	365318	2	1	TRUE	6.29E-05	22.96802	28.95116
rs3523697	G	A	G	A	-0.07056	0.0188	0.12597	0.1533	FALSE	FALSE	FALSE	EmctNB	0.0168	0.263	14731	outcome	TRUE	reported	textfile	27596062	4.22E-08	0.012872	exposure	TRUE	reported	uIS7w6	textfile	365318	2	1	TRUE	8.22E-05	30.04454	28.95116
rs3776299	A	G	A	G	0.044738	0.0014	0.504616	0.4464	FALSE	FALSE	FALSE	EmctNB	0.0118	0.906	14742	outcome	TRUE	reported	textfile	1.43E+08	1.14E-07	0.008438	exposure	TRUE	reported	uIS7w6	textfile	365318	2	1	TRUE	7.69E-05	28.11287	28.95116
rs3918226	T	C	T	C	0.087614	-0.0259	0.069497	0.0874	FALSE	FALSE	FALSE	EmctNB	0.022	0.239	14723	outcome	TRUE	reported	textfile	1.51E+08	9.34E-08	0.01641	exposure	TRUE	reported	uIS7w6	textfile	365318	2	1	TRUE	7.80E-05	28.5052	28.95116
rs4762728	T	C	T	C	-0.03927	0.0051	0.461298	0.3998	FALSE	FALSE	FALSE	EmctNB	0.0137	0.71	12412	outcome	TRUE	reported	textfile	22094469	4.55E-06	0.008565	exposure	TRUE	reported	uIS7w6	textfile	365318	2	1	TRUE	5.75E-05	21.01921	28.95116
rs5589921	G	T	G	T	-0.04993	-0.0135	0.212861	0.1606	FALSE	FALSE	FALSE	EmctNB	0.0161	0.402	14728	outcome	TRUE	reported	textfile	11615048	1.34E-06	0.01033	exposure	TRUE	reported	uIS7w6	textfile	365318	2	1	TRUE	6.40E-05	23.36557	28.95116
rs6214192	A	G	A	G	-0.05956	-0.0093	0.134209	0.1542	FALSE	FALSE	FALSE	EmctNB	0.0164	0.571	14724	outcome	TRUE	reported	textfile	58363552	1.79E-06	0.012471	exposure	TRUE	reported	uIS7w6	textfile	365318	2	1	TRUE	6.24E-05	22.80592	28.95116
rs6219770	A	G	A	G	0.065127	0.0113	0.094864	0.0917	FALSE	FALSE	FALSE	EmctNB	0.0208	0.587	14730	outcome	TRUE	reported	textfile	1.87E+08	4.49E-06	0.014197	exposure	TRUE	reported	uIS7w6	textfile	365318	2	1	TRUE	5.76E-05	21.04333	28.95116
rs6239768	T	C	T	C	0.060788	-0.0028	0.218152	0.1829	FALSE	FALSE	FALSE	EmctNB	0.0153	0.855	14744	outcome	TRUE	reported	textfile	32834232	1.99E-09	0.010133	exposure	TRUE	reported	uIS7w6	textfile	365318	2	1	TRUE	9.85E-05	35.98559	28.95116
rs6248043	G	T	G	T	-0.04125	0.0016	0.369217	0.3449	FALSE	FALSE	FALSE	EmctNB	0.0129	0.901	14296	outcome	TRUE	reported	textfile	1.56E+08	3.21E-06	0.008859	exposure	TRUE	reported	uIS7w6	textfile	365318	2	1	TRUE	5.94E-05	21.6845	28.95116
rs6657811	T	A	T	A	-0.06455	-0.0333	0.101838	0.1237	FALSE	TRUE	FALSE	EmctNB	0.0183	0.0688	14722	outcome	TRUE	reported	textfile	1.09E+08	3.97E-06	0.013994	exposure	TRUE	reported	uIS7w6	textfile	365318	2	1	TRUE	5.82E-05	21.27914	28.95116
rs7137828	T	C	T	C	-0.04505	-0.0366	0.587192	0.5234	FALSE	FALSE	FALSE	EmctNB	0.0134	0.00631	11345	outcome	TRUE	reported	textfile	1.11E+08	1.30E-07	0.008534	exposure	TRUE	reported	uIS7w6	textfile	365318	2	1	TRUE	7.63E-05	27.86993	28.95116
rs7145525	A	G	A	G	0.069868	0.0032	0.107912	0.1314	FALSE	FALSE	FALSE	EmctNB	0.0181	0.86	14729	outcome	TRUE	reported	textfile	53699257	2.09E-07	0.013459	exposure	TRUE	reported	uIS7w6	textfile	365318	2	1	TRUE	7.38E-05	26.94966	28.95116
rs7188578	A	G	A	G	-0.14014	0.0847	0.978151	0.9689	FALSE	FALSE	FALSE	EmctNB	0.0423	0.0452	14296	outcome	TRUE	reported	textfile	81111451	9.06E-07	0.028536	exposure	TRUE	reported	uIS7w6	textfile	365318	2	1	TRUE	6.60E-05	24.11752	28.95116
rs7330686	A	G	A	G	0.054951	-0.0219	0.162465	0.1237	FALSE	FALSE	FALSE	EmctNB	0.0183	0.231	14736	outcome	TRUE	reported	textfile	59150608	1.27E-06	0.011343	exposure	TRUE	reported	uIS7w6	textfile	365318	2	1	TRUE	6.42E-05	23.4685	28.95116
rs736699	G	A	G	A	-0.04675	-0.0082	0.548823	0.6029	FALSE	FALSE	FALSE	EmctNB	0.012	0.494	14739	outcome	TRUE	reported	textfile	26691062	3.16E-08	0.00845	exposure	TRUE	reported	uIS7w6	textfile	365318	2	1	TRUE	8.38E-05	30.60476	28.95116
rs7453657	G	T	G	T	0.065452	-0.0291	0.141565	0.1306	FALSE	FALSE	FALSE	EmctNB	0.0176	0.0982	14296	outcome	TRUE	reported	textfile	1315781	4.41E-08	0.011958	exposure	TRUE	reported	uIS7w6	textfile	365318	2	1	TRUE	8.20E-05	29.95994	28.95116
rs7542713	G	A	G	A	-0.04271	0.0151	0.419214	0.3947	FALSE	FALSE	FALSE	EmctNB	0.012	0.208	14735	outcome	TRUE	reported	textfile	1.69E+08	5.77E-07	0.008545	exposure	TRUE	reported	uIS7w6	textfile	365318	2	1	TRUE	6.84E-05	24.98658	28.95116
rs7570797	G	A	G	A	0.127099	0.0447	0.033586	0.0466	FALSE	FALSE	FALSE	EmctNB	0.0283	0.114	14744	outcome	TRUE	reported	textfile	68645939	5.74E-08	0.02342	exposure	TRUE	reported	uIS7w6	textfile	365318	2	1	TRUE	8.06E-05	29.45054	28.95116
rs7594692	A	G	A	G	-0.05744	0.0332	0.18979	0.1713	FALSE	FALSE	FALSE	EmctNB	0.016	0.038	14296	outcome	TRUE	reported	textfile	10811954	1.43E-07	0.010917	exposure	TRUE	reported	uIS7w6	textfile	365318	2	1	TRUE	7.58E-05	27.68088	28.95116
rs7667549	A	G	A	G	-0.07276	0.0209	0.087328	0.054	FALSE	FALSE	FALSE	EmctNB	0.0273	0.444	14733	outcome	TRUE	reported	textfile	1.07E+08	1.44E-06	0.015098	exposure	TRUE	reported	uIS7w6	textfile	365318	2	1	TRUE	6.36E-05	23.22473	28.95116
rs7692644	T	C	T	C	0.070306	-0.0163	0.08762	0.0647	FALSE	FALSE	FALSE	EmctNB	0.0269	0.545	14296	outcome	TRUE	reported	textfile	35749611	1.94E-06	0.014771	exposure	TRUE	reported	uIS7w6	textfile	365318	2	1	TRUE	6.20E-05	22.65449	28.95116
rs7741818	T	C	T	C	0.120299	-0.0084	0.034374	0.029	FALSE	FALSE	FALSE	EmctNB	0.0397	0.832	14743	outcome	TRUE	reported	textfile	21929487	1.28E-07	0.022778	exposure	TRUE	reported	uIS7w6	textfile	365318	2	1	TRUE	7.63E-05	27.89294	28.95116
rs7750772	T	C	T	C	-0.05989	-0.0349	0.133397	0.1064	FALSE	FALSE	FALSE	EmctNB	0.0204	0.0871	14296	outcome	TRUE	reported	textfile	2.05E+08	1.72E-06	0.012521	exposure	TRUE	reported	uIS7w6	textfile	365318	2	1	TRUE	6.26E-05	22.88159	28.95116
rs7814834	T	C	T	C	0.044849	5.00E-04	0.521659	0.5427	FALSE	FALSE	FALSE	EmctNB	0.0118	0.966	14742	outcome	TRUE	reported	textfile	11530352	1.11E-07	0.00845	exposure	TRUE	reported	uIS7w6	textfile	365318	2	1	TRUE	7.71E-05	28.17365	28.95116
rs7857118	T	A	T	A	0.104171	-0.0177	0.423848	0.4768	FALSE	TRUE	TRUE	EmctNB	0.0117	0.13	14736	outcome	TRUE	reported	textfile	22124141	1.42E-34	0.008494	exposure	TRUE	reported	uIS7w6	textfile	365318	2	1	FALSE	0.000412	150.3927	28.95116
rs969826	A	G	A	G	-0.03967	-0.0029	0.422877	0.4865	FALSE	FALSE	FALSE	EmctNB	0.0119	0.807	14295	outcome	TRUE	reported	textfile	75864627	3.25E-06	0.008523	exposure	TRUE	reported	uIS7w6	textfile	365318	2	1	TRUE	5.93E-05	21.66329	28.95116
rs9799914	A	G	A	G	-0.04639	0.0051	0.498009	0.496	FALSE	FALSE	FALSE	EmctNB	0.0118	0.666	14730	outcome	TRUE	reported	textfile	1.23E+08	3.80E-08	0.008435	exposure	TRUE	reported	uIS7w6	textfile	365318	2	1	TRUE	8.28E-05	30.24702	28.95116

Supplementary Table S3: Characteristics of significant SNPs with genome-wide associations (P<5×10-5) for MMP-1 on LSL

SNP	effect_al	other_all	effect_al	other_all	beta.expo	beta.outc	eaf.expos	eaf.outco	remove	palindrom	ambiguous	id.outcom	se.outcom	pval.outc	outcome	mr_keep.o	pval_orig	data_sour	se.exposu	pval.expo	samplesiz	exposure	mr_keep.e	pval_orig	id.exposu	data_sour	action	SNP_index	mr_keep	samplesize.outcome
rs1073282	A	G	A	G	0.0558	-0.00534	0.3498	0.367796	FALSE	FALSE	FALSE	t3hc6V	0.01634	0.743976	outcome	TRUE	reported	textfile	0.0124	6.80E-06	14742	exposure	TRUE	reported	heG1IZ	textfile	2	1	TRUE	NA
rs1097864	A	T	A	T	0.0649	0.029164	0.7462	0.748458	FALSE	TRUE	FALSE	t3hc6V	0.018855	0.121925	outcome	TRUE	reported	textfile	0.014	3.56E-06	14296	exposure	TRUE	reported	heG1IZ	textfile	2	1	TRUE	NA
rs1099053	T	C	T	C	0.0703	0.016048	0.253	0.234649	FALSE	FALSE	FALSE	t3hc6V	0.01817	0.377118	outcome	TRUE	reported	textfile	0.0135	1.91E-07	14734	exposure	TRUE	reported	heG1IZ	textfile	2	1	TRUE	NA
rs1163502	A	T	A	T	0.0639	0.003141	0.3845	0.377199	FALSE	TRUE	FALSE	t3hc6V	0.016111	0.845408	outcome	TRUE	reported	textfile	0.0142	6.80E-06	10717	exposure	TRUE	reported	heG1IZ	textfile	2	1	TRUE	NA
rs1167312	T	C	T	C	-0.0585	-0.00199	0.296	0.303928	FALSE	FALSE	FALSE	t3hc6V	0.016939	0.906266	outcome	TRUE	reported	textfile	0.0127	4.10E-06	14741	exposure	TRUE	reported	heG1IZ	textfile	2	1	TRUE	NA
rs1168898	A	G	A	G	-0.176	0.017119	0.0455	0.047631	FALSE	FALSE	FALSE	t3hc6V	0.036384	0.637999	outcome	TRUE	reported	textfile	0.03	4.45E-09	14744	exposure	TRUE	reported	heG1IZ	textfile	2	1	TRUE	NA
rs1212203	A	T	A	T	-0.1077	0.011264	0.0741	0.06836	FALSE	TRUE	FALSE	t3hc6V	0.031376	0.71959	outcome	TRUE	reported	textfile	0.024	7.21E-06	14296	exposure	TRUE	reported	heG1IZ	textfile	2	1	TRUE	NA
rs1214179	A	G	A	G	0.0859	0.000344	0.6981	0.695291	FALSE	FALSE	FALSE	t3hc6V	0.017676	0.984475	outcome	TRUE	reported	textfile	0.013	3.90E-11	14296	exposure	TRUE	reported	heG1IZ	textfile	2	1	TRUE	NA
rs1236736	T	G	T	G	0.0861	0.008281	0.1146	0.112742	FALSE	FALSE	FALSE	t3hc6V	0.02499	0.740373	outcome	TRUE	reported	textfile	0.0186	3.67E-06	14744	exposure	TRUE	reported	heG1IZ	textfile	2	1	TRUE	NA
rs1238045	T	C	T	C	-0.0593	-0.02989	0.7098	0.698983	FALSE	FALSE	FALSE	t3hc6V	0.017083	0.080148	outcome	TRUE	reported	textfile	0.0133	8.25E-06	14296	exposure	TRUE	reported	heG1IZ	textfile	2	1	TRUE	NA
rs1417822	A	G	A	G	0.3767	-0.03086	0.9777	0.984415	FALSE	FALSE	FALSE	t3hc6V	0.062952	0.624025	outcome	TRUE	reported	textfile	0.0445	2.56E-17	14731	exposure	TRUE	reported	heG1IZ	textfile	2	1	TRUE	NA
rs1489088	A	G	A	G	-0.2306	-0.01537	0.0214	0.018597	FALSE	FALSE	FALSE	t3hc6V	0.057454	0.789076	outcome	TRUE	reported	textfile	0.0517	8.18E-06	12935	exposure	TRUE	reported	heG1IZ	textfile	2	1	TRUE	NA
rs1498192	A	G	A	G	-0.1601	-0.13153	0.0417	0.02164	FALSE	FALSE	FALSE	t3hc6V	0.054022	0.014904	outcome	TRUE	reported	textfile	0.0351	5.08E-06	14744	exposure	TRUE	reported	heG1IZ	textfile	2	1	TRUE	NA
rs1886263	T	C	T	C	-0.2509	-0.05166	0.0177	0.015988	FALSE	FALSE	FALSE	t3hc6V	0.062367	0.407518	outcome	TRUE	reported	textfile	0.053	2.20E-06	12935	exposure	TRUE	reported	heG1IZ	textfile	2	1	TRUE	NA
rs1891733	T	C	T	C	0.3179	0.104591	0.0131	0.012908	FALSE	FALSE	FALSE	t3hc6V	0.069989	0.135073	outcome	TRUE	reported	textfile	0.0714	8.49E-06	11925	exposure	TRUE	reported	heG1IZ	textfile	2	1	TRUE	NA
rs2155053	T	C	T	C	-0.3212	-0.07619	0.9461	0.93883	FALSE	FALSE	FALSE	t3hc6V	0.032678	0.019728	outcome	TRUE	reported	textfile	0.0264	4.68E-34	14741	exposure	TRUE	reported	heG1IZ	textfile	2	1	TRUE	NA
rs2229629	A	G	A	G	-0.3153	-0.02336	0.0135	0.012473	FALSE	FALSE	FALSE	t3hc6V	0.070308	0.739717	outcome	TRUE	reported	textfile	0.0673	2.80E-06	10887	exposure	TRUE	reported	heG1IZ	textfile	2	1	TRUE	NA
rs2926741	T	C	T	C	-0.0704	0.016193	0.3436	0.367544	FALSE	FALSE	FALSE	t3hc6V	0.016235	0.318576	outcome	TRUE	reported	textfile	0.0126	2.31E-08	14296	exposure	TRUE	reported	heG1IZ	textfile	2	1	TRUE	NA
rs3595909	T	TA	T	TA	0.0698	-0.00558	0.7439	0.735171	FALSE	FALSE	FALSE	t3hc6V	0.017808	0.754213	outcome	TRUE	reported	textfile	0.0154	5.83E-06	11339	exposure	TRUE	reported	heG1IZ	textfile	2	1	TRUE	NA
rs4734879	A	G	A	G	0.1136	-0.00865	0.7422	0.72481	FALSE	FALSE	FALSE	t3hc6V	0.01773	0.625649	outcome	TRUE	reported	textfile	0.0137	1.11E-16	14296	exposure	TRUE	reported	heG1IZ	textfile	2	1	TRUE	NA
rs484915	A	T	A	T	-0.3517	-0.01703	0.5613	0.554868	FALSE	TRUE	TRUE	t3hc6V	0.016285	0.295694	outcome	TRUE	reported	textfile	0.0119	########	14295	exposure	TRUE	reported	heG1IZ	textfile	2	1	FALSE	NA
rs5881124	T	C	T	C	-0.1051	0.036528	0.9266	0.936579	FALSE	FALSE	FALSE	t3hc6V	0.03251	0.261177	outcome	TRUE	reported	textfile	0.0234	7.07E-06	14723	exposure	TRUE	reported	heG1IZ	textfile	2	1	TRUE	NA
rs6717478	A	T	A	T	0.1548	0.062886	0.966	0.963714	FALSE	TRUE	FALSE	t3hc6V	0.041811	0.132566	outcome	TRUE	reported	textfile	0.0338	4.65E-06	14742	exposure	TRUE	reported	heG1IZ	textfile	2	1	TRUE	NA
rs7219205	A	G	A	G	0.0569	-0.01179	0.5023	0.477264	FALSE	FALSE	FALSE	t3hc6V	0.016021	0.461782	outcome	TRUE	reported	textfile	0.0121	2.57E-06	14296	exposure	TRUE	reported	heG1IZ	textfile	2	1	TRUE	NA
rs7277739	A	G	A	G	0.1657	0.096621	0.9671	0.96917	FALSE	FALSE	FALSE	t3hc6V	0.044999	0.031778	outcome	TRUE	reported	textfile	0.0364	5.31E-06	14744	exposure	TRUE	reported	heG1IZ	textfile	2	1	TRUE	NA
rs7709531	T	G	T	G	-0.1416	-0.04033	0.0705	0.074131	FALSE	FALSE	FALSE	t3hc6V	0.029952	0.178159	outcome	TRUE	reported	textfile	0.0238	2.69E-09	14744	exposure	TRUE	reported	heG1IZ	textfile	2	1	TRUE	NA
rs873675	T	C	T	C	0.1234	-0.00068	0.0684	0.057335	FALSE	FALSE	FALSE	t3hc6V	0.033833	0.983888	outcome	TRUE	reported	textfile	0.0234	1.34E-07	14742	exposure	TRUE	reported	heG1IZ	textfile	2	1	TRUE	NA
rs9524612	T	C	T	C	-0.072	-0.00399	0.177	0.179154	FALSE	FALSE	FALSE	t3hc6V	0.020597	0.846553	outcome	TRUE	reported	textfile	0.0162	8.81E-06	14296	exposure	TRUE	reported	heG1IZ	textfile	2	1	TRUE	NA

Supplementary Table S4: Characteristics of significant SNPs with genome-wide associations (P<5×10-5) for ISLSL on

SNP	effect_al	other_all	effect_al	other_all	beta.expo	beta.outc	eaf.expos	eaf.outco	remove	palindrom	ambiguous	id.outcom	pos.outco	pval.outc	se.outcom	outcome	mr_keep.o	pval_orig	data_sour	se.exposu	pval.expo	exposure	mr_keep.e	pval_orig	id.exposu	data_sour	samplesiz	action	SNP_index	mr_keep	samplesize.outcome
rs1012556T	G	T	G	0.073475	-0.0133	0.513407	0.488725	FALSE	FALSE	FALSE	5SY6wc	1.1E+08	0.114336	0.008422	outcome	TRUE	reported	textfile	0.016006	4.42E-06	exposure	TRUE	reported	GCST90200	textfile	7891	2	1	TRUE	NA
rs1042597A	G	A	G	0.227308	-0.01967	0.835107	0.820734	FALSE	FALSE	FALSE	5SY6wc	47881378	0.073728	0.011	outcome	TRUE	reported	textfile	0.021086	4.27E-27	exposure	TRUE	reported	GCST90200	textfile	7891	2	1	TRUE	NA
rs1049016T	C	T	C	0.077418	-0.0049	0.319769	0.357641	FALSE	FALSE	FALSE	5SY6wc	50908336	0.579369	0.008832	outcome	TRUE	reported	textfile	0.016713	3.62E-06	exposure	TRUE	reported	GCST90200	textfile	7891	2	1	TRUE	NA
rs1104585G	T	G	T	-0.19021	0.022683	0.238262	0.172399	FALSE	FALSE	FALSE	5SY6wc	21197755	0.042185	0.011165	outcome	TRUE	reported	textfile	0.017978	3.67E-26	exposure	TRUE	reported	GCST90200	textfile	7891	2	1	TRUE	NA
rs1133911T	A	T	A	0.144942	-0.01875	0.075398	0.030784	FALSE	TRUE	FALSE	5SY6wc	6156417	0.448523	0.024746	outcome	TRUE	reported	textfile	0.029814	1.16E-06	exposure	TRUE	reported	GCST90200	textfile	7891	2	1	TRUE	NA
rs1135137A	G	A	G	-0.16739	0.004129	0.057868	0.024742	FALSE	FALSE	FALSE	5SY6wc	99849162	0.877867	0.026867	outcome	TRUE	reported	textfile	0.033544	6.03E-07	exposure	TRUE	reported	GCST90200	textfile	7891	2	1	TRUE	NA
rs1156899T	C	T	C	0.139496	0.02198	0.086227	0.061345	FALSE	FALSE	FALSE	5SY6wc	1.1E+08	0.214497	0.017707	outcome	TRUE	reported	textfile	0.027913	5.80E-07	exposure	TRUE	reported	GCST90200	textfile	7891	2	1	TRUE	NA
rs1174734T	C	T	C	0.182396	-0.00979	0.050633	0.029563	FALSE	FALSE	FALSE	5SY6wc	49241948	0.695793	0.025036	outcome	TRUE	reported	textfile	0.035245	2.28E-07	exposure	TRUE	reported	GCST90200	textfile	7891	2	1	TRUE	NA
rs1457603C	T	C	T	-0.22187	-0.02992	0.025032	0.038779	FALSE	FALSE	FALSE	5SY6wc	2.21E+08	0.172017	0.021907	outcome	TRUE	reported	textfile	0.049906	8.76E-06	exposure	TRUE	reported	GCST90200	textfile	7891	2	1	TRUE	NA
rs1483903T	C	T	C	-0.39893	-0.01223	0.008349	0.003968	FALSE	FALSE	FALSE	5SY6wc	54881197	0.856514	0.067611	outcome	TRUE	reported	textfile	0.085749	3.28E-06	exposure	TRUE	reported	GCST90200	textfile	7891	2	1	TRUE	NA
rs1490782G	A	G	A	-0.38355	0.041461	0.009615	0.011311	FALSE	FALSE	FALSE	5SY6wc	17443418	0.306214	0.040521	outcome	TRUE	reported	textfile	0.080585	1.94E-06	exposure	TRUE	reported	GCST90200	textfile	7891	2	1	TRUE	NA
rs1705268C	A	C	A	-0.08552	0.006234	0.766937	0.711842	FALSE	FALSE	FALSE	5SY6wc	91179983	0.502661	0.009301	outcome	TRUE	reported	textfile	0.018602	4.28E-06	exposure	TRUE	reported	GCST90200	textfile	7891	2	1	TRUE	NA
rs1892853G	T	G	T	0.082388	-0.00433	0.742532	0.743568	FALSE	FALSE	FALSE	5SY6wc	22309388	0.653695	0.009647	outcome	TRUE	reported	textfile	0.017847	3.91E-06	exposure	TRUE	reported	GCST90200	textfile	7891	2	1	TRUE	NA
rs1896696C	T	C	T	-0.32751	-0.01307	0.011761	0.008391	FALSE	FALSE	FALSE	5SY6wc	97318627	0.78118	0.047036	outcome	TRUE	reported	textfile	0.072574	6.40E-06	exposure	TRUE	reported	GCST90200	textfile	7891	2	1	TRUE	NA
rs2348060C	T	C	T	0.072586	-0.01434	0.359435	0.306239	FALSE	FALSE	FALSE	5SY6wc	1.13E+08	0.116408	0.009133	outcome	TRUE	reported	textfile	0.016201	7.45E-06	exposure	TRUE	reported	GCST90200	textfile	7891	2	1	TRUE	NA
rs2417966G	C	G	C	0.122852	-0.02742	0.248883	0.33848	FALSE	TRUE	FALSE	5SY6wc	21190295	0.002133	0.00893	outcome	TRUE	reported	textfile	0.018008	8.97E-12	exposure	TRUE	reported	GCST90200	textfile	7891	2	1	TRUE	NA
rs4530839C	T	C	T	-0.08747	-0.00031	0.243705	0.21538	FALSE	FALSE	FALSE	5SY6wc	67781475	0.975764	0.010238	outcome	TRUE	reported	textfile	0.018271	1.69E-06	exposure	TRUE	reported	GCST90200	textfile	7891	2	1	TRUE	NA
rs6208649T	A	T	A	0.159898	0.00581	0.055045	0.049353	FALSE	TRUE	FALSE	5SY6wc	4228261	0.77246	0.020094	outcome	TRUE	reported	textfile	0.03496	4.79E-06	exposure	TRUE	reported	GCST90200	textfile	7891	2	1	TRUE	NA
rs6227867G	C	G	C	-0.1547	0.013746	0.059893	0.057708	FALSE	TRUE	FALSE	5SY6wc	1.62E+08	0.448158	0.018123	outcome	TRUE	reported	textfile	0.032913	2.60E-06	exposure	TRUE	reported	GCST90200	textfile	7891	2	1	TRUE	NA
rs7316970A	G	A	G	-0.25193	0.022948	0.022644	0.022685	FALSE	FALSE	FALSE	5SY6wc	35253023	0.419627	0.028434	outcome	TRUE	reported	textfile	0.052355	1.49E-06	exposure	TRUE	reported	GCST90200	textfile	7891	2	1	TRUE	NA
rs7556391C	A	C	A	-0.24239	-0.00924	0.028943	0.062073	FALSE	FALSE	FALSE	5SY6wc	66072134	0.599199	0.017579	outcome	TRUE	reported	textfile	0.046635	2.02E-07	exposure	TRUE	reported	GCST90200	textfile	7891	2	1	TRUE	NA
rs7924462G	A	G	A	-0.14578	-0.003	0.062841	0.045952	FALSE	FALSE	FALSE	5SY6wc	50044512	0.881133	0.020081	outcome	TRUE	reported	textfile	0.032594	7.73E-06	exposure	TRUE	reported	GCST90200	textfile	7891	2	1	TRUE	NA
